# Tomato Leaf Curl New Delhi Virus: An Emerging Virus Complex Threatening Vegetable and Fiber Crops

**DOI:** 10.3390/v9100264

**Published:** 2017-09-21

**Authors:** Enrique Moriones, Shelly Praveen, Supriya Chakraborty

**Affiliations:** 1 Subtropical and Mediterranean Horticulture Institute “La Mayora” (IHSM-UMA-CSIC), Consejo Superior de Investigaciones Científicas, La Mayora Experimental Station, 29750 Algarrobo-Costa, Málaga, Spain; 2 Advanced Center for Plant Virology, Division of Plant Pathology, Indian Agricultural Research Institute, New Delhi 110 012, India; shellypraveen@hotmail.com; 3 Molecular Virology Laboratory, School of Life Sciences, Jawaharlal Nehru University, New Delhi 110 067, India; supriyachakrasls@yahoo.com

**Keywords:** tomato leaf curl New Delhi virus, ToLCNDV, recombination, genetic diversity, control, epidemics, genome structure, host adaptation, DNA satellites, resistance

## Abstract

The tomato leaf curl New Delhi virus (ToLCNDV) (genus *Begomovirus*, family *Geminiviridae*) represents an important constraint to tomato production, as it causes the most predominant and economically important disease affecting tomato in the Indian sub-continent. However, in recent years, ToLCNDV has been fast extending its host range and spreading to new geographical regions, including the Middle East and the western Mediterranean Basin. Extensive research on the genome structure, protein functions, molecular biology, and plant–virus interactions of ToLCNDV has been conducted in the last decade. Special emphasis has been given to gene silencing suppression ability in order to counteract host plant defense responses. The importance of the interaction with DNA alphasatellites and betasatellites in the biology of the virus has been demonstrated. ToLCNDV genetic variability has been analyzed, providing new insights into the taxonomy, host adaptation, and evolution of this virus. Recombination and pseudorecombination have been shown as motors of diversification and adaptive evolution. Important progress has also been made in control strategies to reduce disease damage. This review highlights these various achievements in the context of the previous knowledge of begomoviruses and their interactions with plants.

## 1. Geographical Distribution, Host Range and Epidemics

The tomato leaf curl New Delhi virus (ToLCNDV) is a bipartite begomovirus species (genus *Begomovirus*, family *Geminiviridae*) whose isolates are transmitted in nature by the whitefly *Bemisia tabaci* (order *Hemiptera*, family *Aleyrodidae*) in a circulative and persistent manner [1]. ToLCNDV is an economically important begomovirus reported to cause devastating damage to tomato (*Solanum lycopersicum*) production, and it is more prevalent in northern India [2,3,4,5]. In the last decade, several reports have suggested the spread of ToLCNDV to other vegetable and fiber crops, with the emergence of this virus in the Old World threatening numerous economically important species [3].

ToLCNDV is an important constraint to tomato production in particular, as it causes one of the most predominant and economically important diseases affecting tomato in the Indian sub-continent [3,4,5]. Epidemics of ToLCNDV were limited to Asian countries until recent times, with reported isolates mainly concentrated to India, Pakistan, and Bangladesh in the Indian subcontinent, but they were also found to be present in Thailand, Indonesia, and Taiwan in Southeast and East Asia (Supplementary Materials Table S1). However, in recent years, ToLCNDV has been fast extending its host range and spreading to new geographical regions. From 2012 a westward spread of this pathogen has occurred, with reports from Iran in the Middle East [6] and detection for the first time in the western Mediterranean Basin, with severe disease outbreaks associated with ToLCNDV infections in greenhouse and open-field cucurbit crops of Spain [7]. Moreover, further spread has occurred, with recent reports of ToLCNDV in Tunisia infecting cucurbits (cucumber, *Cucumis sativus*; melon, *Cucumis melo*; zucchini squash, *Cucurbita pepo*) and in Italy, infecting zucchini squash, with isolates closely related to those detected in Spain [8,9]. Sequence analysis suggests that a novel strain of ToLCNDV (ToLCNDV-ES) is spreading in the western Mediterranean region, which seemed to have evolved from the very diverse ToLCNDV population through recombination [7] (see below).

It is well documented that several monopartite begomoviruses are involved in the major disease complexes affecting various crops in the “Old World”. As an exception, ToLCNDV is to date the only bipartite begomovirus that causes diseases in a number of plants belonging to (but not limited to) families including Cucurbitaceae, Euphorbiaceae, Fabaceae, Malvaceae and importantly, Solanaceae [3]. ToLCNDV was initially reported in solanaceous crops, but subsequently many reports of damage to cucurbit crops were also made. In addition to tomato, this virus has been reported as causing damage to relevant vegetable and fiber crop species and also was found to infect a number of weeds. It has been documented that ToLCNDV infects 43 different plant species in Bangladesh, India, Indonesia, Iran, Italy, Malaysia, Pakistan, Sri Lanka, Spain, Taiwan, Thailand, and Tunisia. In 1995, the association of ToLCNDV with tomato leaf curl disease on solanaceous crops was first reported in India [4]. Subsequently, over the period 1996–1998, ToLCNDV was identified in *C. melo, C. sativus, Lagenaria siceraria* and *Luffa cylindrica* from Thailand and in *Solanum nigrum* from Pakistan, in addition to tomato [3,10]. Meanwhile, this virus has spread to various parts of the Indian sub-continent, expanding its host range drastically. Between 2000 and 2010, ToLCNDV has been reported to be associated to infections of various hosts such as *Abelmoschus esculentus*, *Ageratum* spp., *Capsicum annuum*, *Carica papaya*, *Citrullus lanatus*, *C. pepo*, *Cyamopsis tetragonoloba*, *Daucus carota*, *Hibiscus cannabinus*, *Jatropha* spp., *L. siceraria*, *Momordica charantia*, *Solanum melongena*, and *Solanum tuberosum* [11,12,13,14,15]. Further, ToLCNDV was detected infecting *Benincasa hispida*, *Catharanthus roseus*, *Chenopodium album*, *Coccinia grandis*, *Jasminum multiflorum*, *Papaver somniferum*, *Sauropus androgynus* and *Trichosanthes cucumerina*, mainly from India and Pakistan [16,17]. Importantly, the occurrence of ToLCNDV associated with cotton leaf curl disease was reported from Pakistan over the period 2013–2015 [18], which suggests that ToLCNDV, along with several other known cotton-infecting monopartite begomoviruses, could pose a serious threat to cotton cultivation in Pakistan. Fazeli et al. [19] first reported the presence of ToLCNDV along with tomato yellow leaf curl virus (TYLCV) as a mixed infection in *Chrozophora hierosolymitana* and tomato plants from Iran. In subsequent years (2012–2013), ToLCNDV has been found to be associated with a leaf curl disease of cucurbits in Spain [7]. Interestingly, ToLCNDV was also detected causing severe epidemics in, cucumber (*C. sativus*), melon (*C. melo*), and zucchini squash (*C. pepo*) crops of Tunisia [8] and in zucchini squash crops of Italy [9].

As summarized in Supplementary Materials Table S1, full-length DNA-A genomic component sequences are available for isolates of ToLCNDV from cultivated plant species of *Caricaceae*, in papaya (*C. papaya*); Cucurbitaceae including ash gourd (*B. hispida*), bitter gourd (*M. charantia*), bottle gourd (*L. siceraria*), chayote (*Sechium edule*), cucumber, melon, pumpkin (*Cucurbita maxima*), ridge gourd-luffa (*Luffa* sp.), and zucchini squash; Fabaceae, in soybean (*Glycine max*); Malvaceae, including cotton (*Gossypium hirsutum*) and okra (*A. esculentus*); Papaveraceae, in opium poppy (*P. somniferum*); Phyllanthaceae, in sweet leaf (*S. androgynus*); and Solanaceae including eggplant (*S. melongena*), pepper (*Capsicum* sp.), potato (*S. tuberosum*), and tomato. Full-length DNA-A sequences are also available for isolates found present in weeds such as rubber bush (*Calotropis procera*), field bind weed (*Convolvulus arvensis*), lamb’s quarters (*C. album*), nightshade (*S. nigrum*), Santa-Maria (*Parthenium hysterophorus*), and toothed dock (*Rumex dentatus*) [10,12,20,21,22,23]. Whitefly transmission assays have not been conducted for all the previously indicated hosts. Therefore, the understanding of the relevance of specific hosts as inoculum sources during ToLCNDV epidemics will require further study.

In tomato, plants infected with ToLCNDV exhibit typical symptoms including upward or downward curling and crinkling of leaves, vein clearing, yellow mottling, leaf puckering and blistering of leaves. At the later stages of the infection, plants appear stunted with shortened internodes and show impaired fruit setting, leading to complete crop loss [4]. In cucurbits, leaf distortion mosaic diseases are caused by ToLCNDV [6,7,8,9,10]. Satellite DNAs may co-exist with ToLCNDV in natural infections and can modulate its pathogenesis, including its host range (see below). Among DNA satellites, betasatellites are frequently found associated with ToLCNDV infections [24,25]. These DNA satellites depend on the helper virus for their replication and spread, and are known to code for a protein (βC1 protein) that has been associated with pathogenesis probably because of its ability to suppress plant defense mechanisms related to gene silencing [26]. Due to the possible importance of DNA satellites during ToLCNDV infections, a specific section was considered for them in this review.

## 2. Genome Structure and Protein Functions

### 2.1. Viral Genes and Proteins

ToLCNDV is a bipartite begomovirus which contains two similar-sized genomic components (named DNA-A and DNA-B) of ca. 2.8 kb in size. Unlike other bipartite begomoviruses identified in the “New World”, ToLCNDV is a “special” bipartite begomovirus containing an *AV2* gene in its DNA-A component. The DNA-A component has six open reading frames (ORFs) (named AC1, AC2, AC3, AC4, AV1 and AV2) that encode six proteins which help in virus replication, transcription of viral genes, pathogenesis, and its encapsidation. The DNA-B has two ORFs (BC1 and BV1) that encode two proteins involved in the movement of the virus and its gene products [27,28]. DNA-A and DNA-B components share a similar sequence named common region, which aid in replication of DNA-B by a DNA-A-encoded replication initiator protein [27]. The product of the AC1 ORF is the replication initiator protein (Rep), which plays its key role by initiating the rolling circle replication by virtue of its nicking and ligation property. The ORF AC4 is embedded in the *AC1* gene and is characterized as a pathogenicity determinant as well as an RNA-silencing suppressor (RSS) [29]. The ORF AC2 encodes for a 15-kDa protein that in begomoviruses also functions as a pathogenicity factor and RSS [30]. AC3 encodes for a 15.2-kDa protein which is a replication enhancer protein [31]. AV1 codes for coat protein, and AV2 (overlapped to AV1) encodes for a pre coat protein with an additional probable role as RSS [32]. BV1 and BC1 encode a nuclear shuttle protein (NSP) and a movement protein (MP), respectively. ToLCNDV requires the DNA B for symptom development, and the coat protein can be dispensable for systemic infection and symptom development [27]. This established a role for the NSP and MP in systemic infection, as is the case with other bipartite begomoviruses. It has been shown that the NSP of ToLCNDV is a symptom determinant and an avirulence determinant that is the target of host defense responses [33].

Genomic organization and functional attributes of the eight viral proteins of ToLCNDV suggest that most of the proteins are multifunctional. The overlapping and embedded ORFs in the genome tightly regulate expression and function of neighboring proteins. Successful virus establishment in the host is a result of weakening of host defense responses followed by efficient replication, packaging and dissemination. Suppression of plant defenses is essential for virus infection progress and is then treated specifically below.

### 2.2. Suppression of Plant Defenses

**Figure 1:**
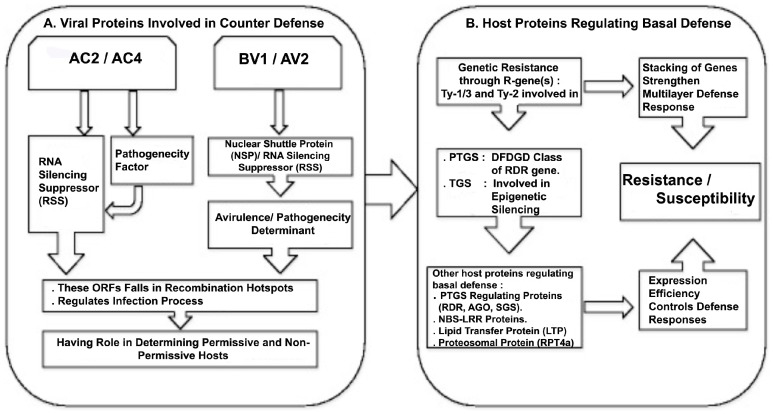
Schematic presentation of cascade of events representing host plant basal defense responses and viral protein-derived counter defense reactions. (**A**) Viral proteins involved in counter defense. AC2 and AC4 are two open reading frames (ORFs) present on the negative strand of DNA-A, coding for proteins with role in RNA-silencing suppression and determination of pathogenicity. BV1 and AV2 ORFs present on the sense strands of DNA-B and DNA-A, respectively, coding for the nuclear shuttle protein (NSP) and an RNA silencing suppressor (RSS), and are characterized as avirulence/pathogenicity determinants. Recombination analysis by Fortes et al. [7] shows that AC2, AC4 and AV1 fall into recombination hotspots, suggesting their fast evolution to counter-defense mechanisms of new hosts; (**B**) host plant proteins regulating basal defense. Functional characterization of genetic resistance based on *Ty*-like genes in tomato suggests their role in regulation of transcriptional and post-transcriptional gene silencing (TGS and PTGS, respectively), stacking of these loci contributes to multilayer defense response. Other basal defense genes induced during infection fall into PTGS-controlling proteins, nucleotide-binding site leucine-rich repeat (NBS-LRR) proteins, a lipid transfer protein (LTP) and the protein of the proteosomal complex. Their relative expression efficiency determines resistance/susceptibility.

Viruses have evolved to encode multifunctional proteins that play key roles in regulating their life cycle as well as suppressing host defenses. Specific defense machineries of the host get activated during viral infection. Resistance genes (R-genes) and RNA interference (RNAi) (or RNA silencing)-mediated defense machineries are two extensively studied responses in plants against viral infection. In tomato, genetic resistance through Ty resistance loci for control of tomato yellow leaf curl disease (TYLCD)-associated viruses has been deployed to contain losses caused by ToLCNDV [34]. Functional characterization of some of these loci suggests their role in strengthening host defense through RNAi [35,36]. To counter host defense, begomoviruses are equipped with proteins with RSS activity that regulate pathogenicity. Viral proteins encoded by ORFs—AC2, AC4, AV2 and BV1 have their role in determining pathogenicity, probably by regulating RNAi, a host basal defense mechanism (Figure 1).

## 3. Genetic Diversity, Host Adaptation and Evolution

### 3.1. Variants of ToLCNDV

The earliest genetic characterization of ToLCNDV corresponds to isolates collected in India and Pakistan during 1990s (e.g., GenBank accession numbers U15015 and Y16421, or AJ620187, respectively, for their DNA-A components). With the advent and general use of advanced molecular techniques such as polymerase chain reaction (PCR), rolling circle amplification (RCA) combined with restriction fragment length polymorphism (RFLP) analysis, and sequencing, the number of full-length genome sequences available for begomoviruses has greatly increased in recent times. In the specific case of ToLCNDV, 136 full-length DNA-A sequences are available in databases as of 10 July 2017 (Supplementary Materials Table S1). As for other begomoviruses and according to the guidelines of the International Committee on Taxonomy of Viruses (ICTV), these DNA-A sequences can be used for taxonomic purposes [37]. Great genetic diversity is found for isolates of ToLCNDV. Thus, based on DNA-A pairwise nucleotide sequence comparisons and criteria for species/strain demarcation currently accepted by the ICTV for begomoviruses (≥91% and ≥94% nucleotide sequence identity for species and strain demarcation, respectively) [37] it was shown by Fortes et al. [7] that most of the ToLCNDV isolates from the Asian locations were in a single group of closely related DNA-A sequences (≥94% identity) despite the high diversity of host species and geographic origin or year of sampling. Also, it was evidenced that ToLCNDV isolates emerging in the western Mediterranean Basin constitute a group of closely related isolates, with DNA-A nucleotide sequence identity greater than 99% among them and less than 94% identity to DNA-As of other ToLCNDV reported so far. Therefore, these isolates belong to a new strain of this virus for which the name “ES” (ToLCNDV-ES) was proposed. Moreover, a number of ToLCNDV isolates from India and Pakistan differed from the reported ones at the strain threshold level (highlighted with asterisks in Supplementary Materials Table S1). Therefore, according to ICTV guidelines, isolates of at least seven different strains are currently present in the population of ToLCNDV that differ genetically (≤94% nucleotide sequence identity) between them and from isolates that belong to the largely predominant ToLCNDV-type group.

**Figure 2:**
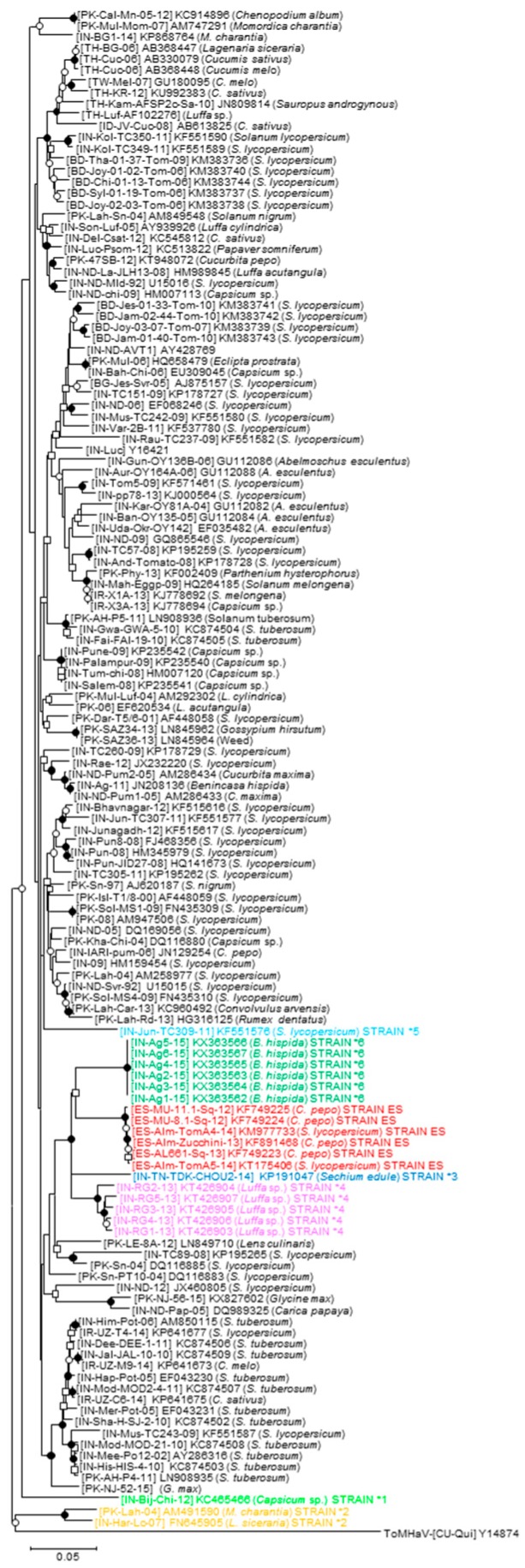
Phylogenetic relationships among DNA-A sequences of the tomato leaf curl New Delhi virus (ToLCNDV) isolates. Phylogenetic relationships among the full-length DNA-A sequences of 136 isolates of ToLCNDV available in the NCBI/GenBank and International Committee on Taxonomy of Viruses (ICTV) databases as of 10 July 2017. The tree was constructed using the neighbor-joining method with the MEGA 5.05 software program (http://www.megasoftware.net). Bootstrap (1000 replicates) analysis was performed and only the nodes with values greater than 50% are labeled, as follows: nodes supported in > 90% and > 70% of bootstrap replicates are marked with filled and open circles respectively, and those with values greater than 50% are marked with open squares. Isolates that differed at the strain threshold level according to criteria for species/strain demarcation currently accepted for begomoviruses by the ICTV [37] (marked with asterisks in Supplementary Materials Table S1) are highlighted with colored letters. Scale bar indicates 0.05 nucleotide substitutions per site. Virus species, GenBank accession number and plant host are indicated for each isolate included in the analysis. The sequence of the DNA-A of isolate (CU-Qui) of the bipartite begomovirus species tomato mosaic Havana virus (ToMHaV) (GenBank accession number Y14874) was included as an outgroup.

As shown in Figure 2, the phylogenetic relationships of the full-length DNA-A sequences of ToLCNDV available in databases as of 10 July 2017 showed that no evident phylogenetic association of isolates was observed in the ToLCNDV population either related with the plant host, the year of sampling, or the geographical origin. There are two isolates (ToLCNDV-(IN-Har-Lc-07) and ToLCNDV-(PK-Lah-04)) in the clade named STRAIN *2 that constituted a topological group in the basal part of the tree robustly separated from all other ToLCNDVs. Interestingly, all ToLCNDV isolates characterized from the western Mediterranean Basin grouped together in a separated topological group, representing a typical case of a founder effect associated with a population bottleneck during the transmission of ToLCNDV to a new area [38,39]. The reduced genetic variation resulting from this bottleneck might have unknown pathological consequences such as compromising the ability of the population to adapt to new selection pressures.

### 3.2. Host Adaption

Differential pathogenicity might be observed for ToLCNDV isolates depending on the host plant. Thus, in a comparative analysis of ToLCNDV pathogenesis performed on diverse solanaceous hosts (pepper (*C. annuum*), *Nicotiana benthamiana*, *Nicotiana tabacum*, and tomato) [40], differential host adaptation was found for the ToLCNDV isolate tested. *N. benthamiana* was found to be the most susceptible host. In contrast, the isolate used was unable to infect pepper, whereas in tomato and *N. tabacum* it induced conspicuous symptoms and accumulated to significantly high titers. The analyses suggested that the co-ordinated expression of host defense responses in each plant species might determine the level of ToLCNDV accumulation and degree of symptom development [40]. Similarly, Fortes et al. [7] showed that isolates of the ToLCNDV-ES strain emerging in the western Mediterranean Basin seem to have adapted to infect cucurbits, but poorly infect tomatoes. The ability of a virus to infect and to induce symptoms in a specific host plant is the outcome of expression of specific sets of genes and dynamic interactions between host and virus-encoded proteins [41]. The ability of ToLCNDV to infect plant species will depend on the genetic factors of the host and the ability of the virus to exploit cellular factors. Coding and non-coding sequences of the genome of begomoviruses might be involved in host adaptation [42,43]. A susceptible host provides all necessary factors required for virus replication, transcription and movement, whereas in a non-permissive host, the virus cannot survive due to incompatible interactions between host and viral factors that restrict viral multiplication and spread. Plants have evolved to activate a coordinated action of several layers of defense versus a pathogen attack. Thus, ToLCNDV infection has been shown to result into the induction of basal defense responses and of genes involved in RNA silencing-mediated defense response [40]. To counteract plant defenses, begomoviruses are known to encode either multiple viral suppressors of RNA silencing [44] or viral determinants that allow for overcoming plant resistance traits (e.g., [45]). The equilibrium between host plant defense response to infection and begomovirus ability to counteract this defense response will determine the success of infection and host adaptation ability. Differential responses to infection might occur in host plants to which a begomovirus is differentially adapted e.g., resistant and susceptible cultivars of the same plant species [46]. In this sense, it is important to note that the ability of viruses to counteract plant defenses might vary from one viral strain to another and even between isolates of the same strain [45]. Limited genetic modifications might be responsible for drastic changes in host adaptation of begomoviruses [47,48]. Also, it should be taken into account that the association of satellite DNAs with ToLCNDV, especially of betasatellites, might alter the pathogenesis of the virus in an individual host plant [24,25,49] or might even help to induce systemic infections and increase its host range as observed in other geminiviruses [50,51]. The latter might be related with the involvement of RNA silencing mechanisms in plant defenses against ToLCNDV [52] and the help of the strong suppressor of gene silencing present in betasatellites [26]. Therefore, host adaptation of ToLCNDV might also be dependent on the presence of betasatellite collaborator DNAs. The role of satellite DNAs in ToLCNDV infections will be discussed in a specific section of this review.

### 3.3. Recombination and Pseudo-Recombination as Motors of Diversification

**Figure 3:**
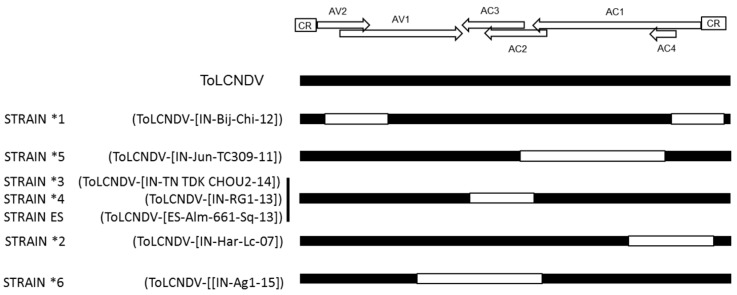
Putative recombination events of isolates of ToLCNDV, representative of different strains. Schematic representation of the putative recombination events present in the DNA-A of isolates of tomato leaf curl New Delhi virus (ToLCNDV) that differ with reported ones at the strain threshold level compared to ToLCNDV-type isolates and deduced based on the results from recombination detection software program (RDP4; Version 4.0, http://web.cbio.uct.ac.za/~darren/rdp.html). Black color indicates ToLCNDV-derived sequences; white color indicates sequences derived from other begomovirus species (adapted and actualized from Fortes et al. [7]).

In field studies, ToLCNDV isolates have been identified in mixed infections along with isolates of several other bipartite begomovirus species such as bhendi yellow vein mosaic virus, chilli leaf curl virus, cotton leaf curl virus, several tomato-begomoviruses, and betasatellites [11,15,18,53]. Genetic recombination allows parental viruses present in mixed infections to exchange genetic information and derive it to their progeny in a parasexual reproduction manner; this mechanism is a key process in the evolution of many virus families and has been extensively recorded for members of the family *Geminiviridae* [54]. Recombination exchanges at the intraspecies and interspecies level are frequent in begomoviruses. Analyses of ToLCNDV sequences suggested that recombination drives the evolution of this virus. Thus, as pointed by Fortes et al. [7], a recombinant origin is suggested for ToLCNDV isolates that differ from others at the strain threshold level (Figure 3). It is important to note, that as observed for other virus groups [55], adaptive recombination in geminiviruses might be extremely efficient [56]. Thus, significant changes in host adaptation can be observed in recombinant begomoviruses [57] even resulting in severe epidemics [58], suggesting recombination as a mechanism to evolve and adapt to changing environmental conditions.

Recombination sites in DNA-A of ToLCNDV suggest that regions having ORFs AC2, AC4 and AV2 are more prone to recombination (Figure 3). Interestingly viral proteins coded by these ORFs have roles in pathogenicity and RSS activity. The evolved recombinant strain ToLCNDV-ES is adapted to infect cucurbits (zucchini squash, cucumber, melon) with poor adaptation for infecting tomato, unlike ToLCNDV isolates reported in India [7]. This might suggest that viral counter defense strategy is host-dependent, with ToLCNDV-ES strain evolved from ToLCNDV isolates of Asian origin and adapted to infect cucurbits.

During mixed infections of ToLCNDV with other bipartite begomoviruses, exchange of genomic components might occur leading to the pseudo-recombination phenomenon. The effect of pseudo-recombination between the components of ToLCNDV and those of isolates of the tomato-infecting bipartite begomovirus tomato leaf curl Gujarat virus (ToLCGV) on the viral pathogenesis was first demonstrated experimentally by Chakraborty et al. [59] showing that supervirulent viruses with enhanced pathogenicity might derive from pseudo-recombination. Similar supervirulence has also been shown for the pseudo-recombination of ToLCNDV with isolates of the bipartite begomovirus tomato leaf curl Palampur virus [60]. Further, trans-replication involving ToLCNDV and isolates of a tomato-infecting monopartite begomovirus (tomato leaf curl Ranchi virus) and its associated betasatellite was also shown [61] which can determine novel phytopathological characteristics.

## 4. Biology and Interaction of Satellite Molecules with ToLCNDV

Similar to “Old World” monopartite begomoviruses, ToLCNDV has also been found to be associated with DNA satellite molecules in field conditions [11,18,62,63]. Associated satellites can be alphasatellites and betasatellites [1]. The association of an alphasatellite with tomato leaf curl disease was observed in Pakistan [64]. Recently, a cotton leaf curl disease-associated alphasatellite has been isolated from plants affected with tomato leaf curl disease in the Varanasi region of India [62]. Simultaneous association of ToLCNDV with alphasatellite and betasatellite molecules has also been reported [49]. The biology and interaction of satellite molecules with ToLCNDV has been investigated; however, scarce information is available about the frequency of these molecules in ToLCNDV epidemics.

### 4.1. Disease Severity

The association of isolates of the betasatellite (chilli leaf curl betasatellite) (ChLCB) with ToLCNDV helper component (DNA-B) and isolates of the bipartite begomovirus pepper leaf curl Lahore virus was first reported from chilli leaf curl disease-affected plants from Pakistan [65]. The biology of the interaction between ToLCNDV (DNA-A and DNA-B) and ChLCB was studied on *N. benthamiana* plants [24], showing that enhanced symptoms occurred. Recent studies indicated the presence of ToLCNDV, with several monopartite begomovirus-betasatellite complexes infecting chilli plants in India [11] with betasatellites modulating infections. Moreover, Jyothsna et al. [25] identified ToLCNDV in mixed infections with isolates of cotton leaf curl Multan betasatellite, tomato yellow leaf curl Thailand betasatellite (TYLCTHB) and papaya leaf curl betasatellite from tomato plants, and also found isolates of bhendi yellow vein betasatellite and papaya leaf curl betasatellite from potato plants in field conditions. In the case of betasatellites, the study suggested that enhanced level of helper virus components could occur in these interactions and that antagonistic interaction between ToLCNDV DNA-B and betasatellites occur. Isolates of Luffa leaf distortion betasatellite were found along with ToLCNDV from several plant species such as cucumber, *L. sicerania*, *L. cylindrica* and *M. charantia*, and their effect on modulation of ToLCNDV pathogenesis on *N. benthamiana* plants was demonstrated. Recently, ToLCNDV and TYLCTHB have been reported to cause severe leaf curl disease of potato in Pakistan [21]. Also, ToLCNDV was identified alongwith a cotton leaf curl virus–cotton leaf curl Multan betasatellite complex from cotton fields in Pakistan [18]. Altogether, mixed infections of ToLCNDV with other begomovirus species and betasatellites have apparently resulted in enhanced disease severity in the infected plants.

Trans-replication of diverse betasatellites by ToLCNDV DNA-A was demonstrated in *N. benthamiana* and tomato plants, and the results suggest host-specific interactions in the trans-replication process [66]. Mixed infection of ToLCNDV and ToLCGV, along with isolates of tobacco leaf curl betasatellite has been reported in natural infections of a resistant chilli cultivar. The presence of DNA-B and betasatellites leads to severe disease damage as compared to the complex lacking betasatellite [53]. Association of betasatellites with ToLCNDV DNA-A (in the absence of DNA-B) also emphasizes that this betasatellite could substitute the movement function of the DNA-B component.

### 4.2. Extending Host Range

Initial observation of the natural co-existence of ToLCNDV DNA-A with DNA-B component on tomatoes was done in India. In subsequent years, ToLCNDV was observed to infect plant species belonging to Cucurbitaceae, Caricaceae, and Malvaceae in addition to plant species belonging to the Solanaceae family. Studies indicated association of betasatellite molecules in the majority of these interactions, suggesting the involvement of these molecules to widen the host range of the virus. Association of ToLCNDV with betasatellite helps in the systemic spread of the virus in the host plant and also enhances virus accumulation. This suggests that ToLCNDV might profit from the adoption of betasatellites found during mixed infection along with other begomoviruses in a permissive host. Subsequently, ToLCNDV had maintained betasatellite as it might enhance its survival fitness. Singh et al. [67] demonstrated that the interaction between ToLCNDV and betasatellites such as isolates of radish leaf curl betasatellite and croton yellow vein mosaic betasatellite lead to leaf curl disease on tomato and radish, reflecting role of betasatellite contributing to host range of ToLCNDV. Similarly, association of ToLCNDV with DNA-B and betasatellite of other begomoviruses led to breakdown of natural resistance in chillies [53]. Collectively, these data emphasize that ToLCNDV effectively utilizes mixed infections, among others with DNA satellites, as a means to adapt to several hitherto non-hosts.

## 5. Virus–Vector Interactions

Under natural conditions ToLCNDV is transmitted by the whitefly *B. tabaci*. This virus is known to be transmitted by several cryptic species of the *B. tabaci* complex [68]. *B. tabaci* species Asia 1, Asia II 1/5/7 and Middle East-Asia Minor 1 are known to transmit this virus in different south Asian regions. In contrast, different *B. tabaci* species seem to be spreading ToLCNDV in the western Mediterranean region. The Mediterranean Q1 cryptic species has been found to be spreading ToLCNDV in southern Spain in different crops such as tomato, zucchini, and melon. Seven different haplotypes belonging to the Mediterranean-Q1 cryptic species were found to be associated with ToLCNDV infections based on partial mitochondrial cytochrome oxidase I sequences [69].

Nariani and Vasudeva [70] performed initial studies on the transmission of ToLCNDV and determined the transmission characteristics with respect to its whitefly vector. Butter and Rataul [71] completed studies about the virus–vector relationship, showing a persistent circulative transmission manner. Even a single whitefly can transmit the virus and females possess higher transmission efficiency compared to males. Whiteflies require a minimum of 30 min of acquisition access time to transmit the virus. However, increasing acquisition and inoculation access times result in higher transmission efficiencies. It was observed that with an acquisition access time of 24 h, male and female whiteflies retained their infectivity for long duration.

As for other begomoviruses, scarce information is available for ToLCNDV regarding the interactions with the whitefly vector. Evidence has suggested that intimate molecular and cellular interactions exist between begomovirus genes and proteins and that of the whitefly and that crucial protein receptors are present in the gut of *B. tabaci* that allow virus entry into the insect haemolymph [72]. Recent studies conducted by Rana et al. [73] suggested the involvement of a protein named as midgut protein to facilitate transport of ToLCNDV from the digestive tract to the haemolymph.

## 6. Control Strategies to Reduce Disease Damage

### 6.1. Management Practices

Human activity combined with global warming are providing conditions that favor the emergence of begomoviruses including ToLCNDV epidemics, among others. This is the cause, for example, of the recent spread of the latter virus from Asia to several countries of the Mediterranean Basin causing severe epidemics in cucurbit crops [7]. Much experience in begomovirus epidemic management has been accumulated in the last years, especially for the widely distributed tomato yellow leaf curl disease caused by tomato yellow leaf curl-like begomoviruses [74]. There is an agreement in that effective control of begomoviruses as for other plant viruses should rely on integrated management approaches by means of a combination of diverse management methods that are cost effective and cause the least damage to the environment [75]. Administrative, legal and technical practices should be then combined in a coordinated manner to prevent ToLCNDV emergence and limit crop losses. Management of ToLCNDV solely based on the chemical control of the vector *B. tabaci* will be difficult and probably ineffective. Moreover, the intensive use of insecticides might result in the emergence of *B. tabaci*-resistant populations and may cause severe environmental damage. Nevertheless, chemical control can help to contain disease spread in certain conditions as shown for other begomoviruses [76]. Also, biological control of the insect vector might be considered to reduce insect vector infestation and limit disease spread, although confirmation studies are needed in target conditions [77]. Understanding the epidemiology of the disease and the factors that favor emergence of ToLCNDV in a specific situation is essential to design effective management approaches to minimize the disease damage. Since ToLCNDV is evolving very fast and is spreading to new regions, developing new strains that extend their ability to infect new hosts, no single strategy will work to contain losses caused by this virus. The first line of defense is the avoidance of the dispersal of the virus to a disease-free region. For this, control of plant commercial trade (including planting material) is essential, reinforcing plant quarantine measures. After the onset of ToLCNDV viral epidemics, cultural control strategies can be adopted that might be effective to reduce virus sources and disease pressure. Some of these strategies include crop placement in time and/or space in order to avoid periods of high whitefly populations, the use of virus-free planting material, elimination of virus sources at the early beginning of the epidemic, the use of physical barriers between flying whiteflies and the crop host especially in protected crops, and discontinuous cropping to break the disease cycle, etc. As for most plant viruses, the best and most effective management approach to control begomoviruses such as ToLCNDV is the use of genetic resistance in the host plant if available [78]. Inclusion of cultivars with durable resistance within an integrated management approach is very interesting, because it can confer effective protection without additional labor, reducing environment damage. Thus, intensive research is being carried out to produce cultivars showing increased virus resistance to ToLCNDV either by means of classical breeding or by using transgenic approaches, which are summarized specifically below. However, it should be taken into account that sustainable control of ToLCNDV solely based on the continuous use of resistance might be compromised by the tendency of viruses to evolve and overcome resistant traits (e.g., [79]). Therefore, the combination of virus resistance with other control practices in integrated management approaches might help to minimize the risk of resistance break for more durable control [80]. It is worth mentioning that the use of insect resistance in the host plant is also an attractive alternative for reducing the pressure of insect-transmitted viruses which has been demonstrated to be effective in limiting begomovirus spread [81]. Host resistance to *B. tabaci* can be combined with other methods of insect control. Moreover, resistance against *B. tabaci* will complement resistance against the viruses it transmits and help to reduce the insecticide applications required to minimize insect populations. The limitation of whitefly pressure through structures management combined with the development of resistance traits (see below) and biological control [82] are resulting in promissory control of the recent ToLCNDV epidemics in protected cucurbit crops in southern Spain.

### 6.2. Host Plant Resistance

Plants have evolved a coordinated action of multilayered defense mechanisms, which are activated during infection. Permissive and non-permissive host interactions for a particular viral infection are dependent on the effective confrontation between host defense and viral counter defense responses. Host-specific induction and expression efficiency of genes coding for proteins involved in basal defense mechanism determine plant resistance or susceptibility to a particular viral infection (Figure 1). Besides this, due to constant pressure exerted by the host plants, viruses have evolved to encode multifunctional proteins that play key roles in regulating their life cycle as well as suppressing host defenses, in order to extend host range. Kushwaha et al. [40] have shown that ToLCNDV infection induces host-specific expression of genes involved in defense such as basal host defense responses including nucleotide-binding site and leucine-rich repeat (NBS-LRR) proteins, post-transcriptional gene silencing machinery (RNA-dependent RNA polymerase 6, RDR6; argonaute 1, AGO1; and suppressor of gene silencing 3, SGS3) and a lipid transfer protein (LTP). Also, another host protein from the 26S proteasomal subunit RPT4a (SlRPT4), binds to the intergenic region (IR) of ToLCNDV DNA-A and DNA-B and inhibits the binding of RNA Pol-II for bi-directional transcription of the viral genome [83]. Expression efficiency of these key genes in different plant hosts determines the level of ToLCNDV infection. On the other hand, recombination patterns in this virus (see above) [7] suggest the evolution of viral genomes, confined largely in the regions coding for suppressors of RNA silencing and pathogenicity factors. Hence host–virus interactions regulating gene expressions determine permissive and non-permissive hosts.

Breeders have exploited natural source of resistance against viral infection for many years. Tolerance/resistance traits against ToLCNDV are available in wild tomato relatives [84], cucurbits [85], potato [46], and *Luffa* sp. [86,87]. Mapping studies of tomato and cucurbit genotypes led to the identification of different resistance/tolerance genes, which are being used for developing resistance against ToLCNDV [85,88]. In tomato, six resistance loci have been deployed to build resistance against different variants of tomato leaf curl viruses. These resistance loci (dominant genes *Ty-1*, *Ty-2*, *Ty-3*, *Ty-4* and *Ty-6*; recessive gene *ty-5*) have different origins; *Ty-1*, *Ty-3 Ty-4* and *Ty-6* are introgressed from different accessions of *Solanum chilense*; *Ty-2* is from *Solanum habrochaites*; and *ty-5* is from *Solanum peruvianum* [89,90]. Out of these six resistance/tolerance loci, *Ty-1*, *Ty-2* and *Ty-3* are commonly used. The resistance loci *Ty-1* and *Ty-3* are present on chromosome 6 and are allelic [36]. They belong to the DFDGD catalytic motif class of RDR genes, which are involved in the biogenesis and amplification of small interfering RNA (siRNA) required for transcriptional gene silencing (TGS) by AGO4-mediated siRNA-directed DNA methylation (RdDM) [35]. Infection by isolates of the monopartite begomovirus species tomato yellow leaf curl virus (TYCV) up-regulates 70 genes in a tomato line, with *Ty-2* having introgressed. Among them, the silencing of three genes affecting expression of a lipocalin-like protein, a permease-I-like protein and a hexose transporter partly compromised the resistance [91,92]. Yang et al. [93] proposed the involvement of the *Ty-2* gene in mediating epigenetic silencing. These findings suggest that instead of classical R gene-mediated hyper-sensitive response (HR), these Ty loci seem to adopt a different mechanism of strengthen host defense response. Prasanna et al. [34,94] have shown that the combination of *Ty-2* and *Ty-3* genes enhanced the resistance level of tomato lines against ToLCNDV and betasatellite.

### 6.3. Genetic Engineering Approaches

Genetic engineering-based development of pathogen derived resistance (PDR) was initiated in late 1990s for tomato leaf curl viruses. Various approaches have been used to target key viral genes, which affect the vital functions of virus establishment. Sense/antisense and RNAi-based silencing of viral genes has been deployed for developing transgenic plants with resistance to ToLCNDV. Different viral genes were targeted to develop different degrees of virus resistance. Coat protein (*AV1*) and pre-coat protein (*AV2*) genes had been used as target for development of resistance either by using them in sense orientation [95,96] or by means of designing artificial microRNAs [32]. Rep protein encoded by *AC1* was targeted in antisense orientation [97,98]. Truncated Rep with sequences from the *AC4* gene was used to target two viral genes using a different RNAi-mediated approach [99]. *AC2* and *AC4* were also targeted using an artificial trans-acting small interfering RNA approach [100]. Transgenic resistance developed in tomato for different variants of ToLCNDV; using different strategies seems to be a promising approach.

## 7. Concluding Remarks

ToLCNDV is arguably the most destructive epidemic-causing bipartite begomovirus in the Indian sub-continent and it has recently spread to other regions. Emergence of ToLCNDV epidemics has led to increased yield losses in tomato and other economically important crops. As yet, limited knowledge is available about ToLCNDV-associated viruses and their epidemics, which is essential to helping the design of novel control approaches. Conventional control practices alone provide far from adequate control of ToLCNDV. However, the integration of several of these practices following recommendations based on an appreciation of the epidemiology of the disease may facilitate management of ToLCNDV epidemics. Therefore, integrated management strategies combining several control practices are recommended. However, more knowledge about the epidemiology and ecology of this complex disease is needed to develop better control strategies. Ideally, in an increasingly unstable climate, which will have profound effects upon virus vectors, we should be able to predict the nature and variation of ToLCNDV epidemics as this virus seems to exhibit a more rapid response time than delivery of control alternatives. Also, more knowledge about ToLCNDV diversity and factors driving the evolution of its populations is essential to establishing more effective and durable measures to control epidemics. Good progress has been made in developing ToLCNDV-resistant tomato and cucurbit cultivars in past years, but much work should be done for other affected hosts. These developments, and the combination of traditional and marker-assisted breeding techniques, will result in improved cultivars for growers. In the area of resistance, research has divided its efforts between building a fundamental knowledge base and responding reactively to real agronomic problems, the latter being delivered where possible to the agricultural industry, usually in the form of markers or tools to help rapid advance in breeding programs. However, limited knowledge about pathogenesis mechanisms of ToLCNDV-associated viruses and the precise interactions with satellites or the *B. tabaci* vector hinder our ability to develop novel and definitive control strategies. Much research should be done to solve the latter limitations. Extremely complex populations of ToLCNDV exist, with variants exhibiting variable host ranges and some of them better adapted to infect specific host plants. Understanding the basic mechanisms associated with such a host-adaptation will provide novel tools to protect plants against infections. Active research should be done in this sense in the near future. In the current social climate the use of resistances based upon genetically modified (GM) plants still will be limited and rather control strategies will exploit diverse natural resistance sources. However, GM approaches based upon gene silencing have provided extremely powerful opportunities for resistance strategies against plant viruses and its potential might not be ignored in the longer term. An effective alternative for specific and durable resistance may be to combine natural and GM resistance emerging from increasing molecular understanding of ToLCNDV–plant host/vector interactions.

## Supplementary Materials

Supplementary materials can be found at www.mdpi.com/1999-4915/9/10/264/s1.
